# The cAMP sensors, EPAC1 and EPAC2, display distinct subcellular distributions despite sharing a common nuclear pore localisation signal

**DOI:** 10.1016/j.cellsig.2015.02.009

**Published:** 2015-05

**Authors:** Euan Parnell, Brian O. Smith, Stephen J. Yarwood

**Affiliations:** Institute of Molecular, Cell and Systems Biology, College of Medical, Veterinary and Life Sciences, University of Glasgow, G12 8QQ, UK

**Keywords:** EPAC, exchange protein activated by cAMP, GEF, guanine nucleotide exchange factor, cAMP, EPAC, Targeting, Nucleus

## Abstract

We have identified a conserved nuclear pore localisation signal (NPLS; amino acids 764–838 of EPAC1) in the catalytic domains of the cAMP-sensors, EPAC1 and EPAC2A. Consequently, EPAC1 is mainly localised to the nuclear pore complex in HEK293T cells where it becomes activated following stimulation with cAMP. In contrast, structural models indicate that the cAMP-binding domain of EPAC2A (CNBD1) blocks access to the conserved NPLS in EPAC2A, reducing its ability to interact with nuclear binding sites. Consequently, a naturally occurring EPAC2 isoform, EPAC2B, which lacks CNBD1 is enriched in nuclear fractions, similar to EPAC1. Structural differences in EPAC isoforms may therefore determine their intracellular location and their response to elevations in intracellular cAMP.

## Highlights

• EPAC isoforms, EPAC1, EPAC2A and EPAC2B are differentially localised in cells.
• EPAC1 is localised to the nuclear pore complex.
• Nuclear localisation of EPAC1 is through a conserved catalytic region.
• The CNBD1 domain in EPAC2A interferes with nuclear targeting.

## 1 Introduction

Cyclic adenosine monophosphate (cAMP) signalling is initiated in response to Gs-coupled protein receptor (GPCR) activation at the cell membrane. The subsequent dissociation of G-protein alpha subunits leads to adenylate cyclase (AC) activation. The conversion of ADP to cAMP by AC leads to a rise in intracellular cAMP and activation of the effector proteins, protein kinase A (PKA), exchange proteins activated by cAMP (EPAC) and cyclic nucleotide gated ion channels. The cAMP signal is terminated by the cAMP phosphodiesterase (PDE) family [1], which catalyses the conversion of cAMP into 5′-AMP. The intracellular localisation of PDEs allows the formation of distinct subcellular pools of cAMP, which selectively activates co-distributed EPAC and PKA molecules. Therefore, cAMP signalling is not only regulated by the induction and depletion of the cAMP signal, but also by the localisation of effector molecules [2].

The compartmentalisation of PKA activity has been extensively studied [3–5], but the effects of EPAC localisation on cAMP signalling are only beginning to be appreciated. EPAC proteins are a family of cAMP-activated guanine nucleotide exchange factors (GEFs) for the small Ras-like GTPases, Rap1 and Rap2 [6,7]. The first clues that indicated a role for compartmentalisation in the regulation of EPAC signalling arose during the study of its redistribution during the cell cycle. During interphase EPAC1 was observed to adopt a perinuclear distribution [8] and, as the cell cycle progressed, EPAC underwent translocation, co-localising with microtubules, the mitotic spindle and the contractile ring [8]. Subsequently, functional importance has been ascribed to the direct interaction of EPAC1 with microtubules [9] and microtubule accessory proteins [10–13]. EPAC1 has been shown to stabilise microtubule polymerisation [9] and promote actin stability within vascular endothelial cells (VECs) [12,14]. Thus the distribution of EPAC within the cell appears to strongly correlate with the nature of the cellular effects induced in response to cAMP.

Three EPAC isoforms, EPAC1, EPAC2A and EPAC2B display different cellular distributions, tissue expression and physiological roles in humans [15]. Recent studies have focused on the nuclear localisation of EPAC1, which occurs during interphase [8,16–19]. Nuclear localisation is mediated by an interaction between the zinc finger domain of the nuclear pore protein RanBP2 (Ran Binding Protein 2) and the catalytic domain of EPAC1 [17]. Interestingly, this interaction limits the GEF activity of EPAC1, suggesting that complex formation may negatively regulate EPAC activity within the cell. Given the growing appreciation that compartmentalisation of EPAC proteins controls their function and activity; we aim here to investigate the structural elements in EPAC isoforms that facilitate their recruitment to the nuclear membrane.

## 2 Materials and methods

### 2.1 Materials

Forskolin and rolipram were purchased from Millipore, Hertfordshire, UK. RedDot nuclear stain was purchased from Cambridge Bioscience, Cambridge, UK. Protein A/G beads were from GE Healthcare, Buckinghamshire, UK.

### 2.2 Constructs

pFlag-CMV2-EPAC2A and pFlag-CMV2-EPAC2B were kind gifts from Professor Susumu Seino, Kobe University, Japan [15]. pMT2-HA-EPAC1 and pMT2-HA-EPAC2 were generously provided by Professor Johannes Bos, University of Utrecht, Netherlands [6,20]. Generation of pFlag-CMV2-EPAC1-620–881, pFlag-CMV2-EPAC1-691–881, pFlag-CMV2-EPAC1-764–881, and pFlag-CMV2-EPAC1-838–881 has been described previously [10].

### 2.3 Mutagenesis

Site directed mutagenesis was done using the Quikchange Mutagenesis kit from Agilent. pFlag-CMV2-EPAC1 was mutated to pFlag-CMV2-EPAC1-R805N-A806T-M809T (Area1, F-GATGAGAATGATGGCCGACACCGCGCGGACCCTGCACCACTGCCG, R-CGGCAGTGGTGCAGGGTCCGCGCGGTGTCGGCCATCATTCTCATC), pFlag-CMV2-EPAC1-Δ824–844 (Area2, F-CGAGTTTCCCACCTCCCAGCCAGCACCTGGG, R-GCCCAGGTGCTGGCTGGGAGGTGGGAAACTCG), pFlag-CMV2-EPAC1-Δ764–838 (F-CGACTGGCCAGGATTTCCACATGC, R GCATGTGGAAATCCTGGCGAGGGCCAGTCG) and pFlag-CMV2-EPAC1-P819A-P821A-P824A (3P-A, F-GCCGAAGCCACAACGCGGTGGCGCTCTCAGCGCTCAGAAGCCGAGTTTCC, R-GGAAACTCGGCTTCTGAGCGCTGAGAGCGCCACCGCGTTGTGGCTTCGGC), respectively.

### 2.4 Antibodies

Anti-EPAC1 (Clone 5D3), phospho-CREB (Ser 133) and normal rabbit IgG were from New England Biolabs (Ipswich, UK). Anti-Ran GTPase was purchased from BD signal Transduction, Oxford, UK. Anti-RanBP2 (#2938 for western blot analysis and #6429 for immunofluorescent detection) was purchased from Abcam, Cambridge, UK. Anti-HA, Anti-Flag (clone M2) and normal mouse IgG were from Sigma Aldrich, Dorset, UK. Alexa-Fluor secondary antibodies anti-rabbit/anti-mouse 488 nm and 568 nm were purchased from Invitrogen, Paisley, UK. Near-Infrared (IR) secondary antibodies were from Licor Biosciences (Nebraska, USA; anti-rabbit/anti-mouse 680 nm and 700 nm).

### 2.5 Cell culture

Stably transfected Human Embryonic Kidney (HEK293T) cells, expressing either 3xFlag-myc-CMV-26 vector (Sigma-Aldrich, UK) containing full-length human EPAC1 or vector alone, were prepared by Dundee Cell Products (Dundee, UK). HEK293T cells were grown in Dulbecco's modified Eagle's medium (DMEM) 10% (v/v) foetal bovine serum (Sigma-Aldrich, UK), 2% (v/v) glutamine (Sigma-Aldrich, UK) and 2% (v/v) penicillin/streptomycin (Sigma-Aldrich, UK) and incubated at 37 °C in 5% (v/v) CO_2_. Selection of stable cell lines was maintained by addition of 400 mg/ml G418 (Sigma-Aldrich, UK) to growth medium.

### 2.6 Immunofluorescent confocal microscopy

Cells were seeded at a density of 1 × 10^5^ on ethanol sterilised 13 mm glass coverslips and allowed to adhere overnight. Cells were then transfected using Lipofectamine 2000 transfection reagent (Invitrogen), according to manufacturer's guidelines. Cells were then stimulated with indicated treatments prior to fixation with Fixing Buffer (3% (w/v) paraformaldehyde, 1% (w/v) sucrose, 1 mM CaCl_2_, 1 mM MgCl_2_ in PBS (37 mM NaCl, 2.7 mM KCl, 8 mM Na_2_HPO_4_, 1.46 mM KH_2_PO_4_, pH 7.4)). Coverslips were then quenched for 10 min in 50 mM NH_4_Cl in PBS, permeabilised for 4 min with 0.1% (v/v) Triton X-100 in PBS. Cells were blocked with 0.02% (v/v) goat serum in 0.02% (w/v) fish skin gelatine in PBS, filtered (0.2 μm Nalgene vacuum filter). Primary and secondary antibodies (anti-mouse/anti-rabbit FITC/Rhodamine conjugates or Rhodamine-Phalloidin in actin stained cells) were then incubated for 1 h at room temperature (RT) sequentially before treatment with 4′, 6-diamidino-2-phenylindole (DAPI, 10 μg/ml) for 20 min at room temperature. Coverslips were washed in 3 × in Block buffer between incubations. Coverslips were then mounted onto glass slides using Shandon Immuno-Mount (Thermo Fisher Scientific, UK) and analysed using a 63X Zeiss oil immersion objective, on a Zeiss confocal microscope (Carl Zeiss, Germany) equipped with a Zeiss LSM5 Pascal instrument.

### 2.7 Immunoprecipitation

Cells were grown to 90% confluency and then lysed in immunoprecipitation (IP) buffer Hepes pH 7.4, 150 mM NaCl, 5 mM EDTA, 1 mM NaF, 10 mM NaPO4, 1% (w/v) Triton X-100 plus protease inhibitor cocktail (Roche). Following lysis, cell debris was removed by centrifugation (10,000 rpm, 10 min). Lysates were then pre cleared with normal mouse IgG and IP carried out with anti-EPAC1 (5D3) or anti-Flag antibodies and then incubated with protein A or protein G beads, respectively, for 1 h (4 °C, rotating). Beads were collected and washed 3 times and then boiled in electrophoresis buffer (63 mM Tris–HCl, 10% (v/v) glycerol, 2% (w/v) SDS, 0.0025% (w/v) bromophenol blue). Cell lysates and IP samples were analysed by western blotting.

### 2.8 Fractionation and western blotting

Cells were fractionated using a nuclear fractionation kit (Active Motif) into cytoplasmic and nuclear components. Equal amounts of lysate protein were then separated on 7% and 12% (w/v) SDS PAGE gels, transferred to nitrocellulose membranes and then incubated with shaking for 1 h in block buffer (1% (w/v) skimmed milk powder in TBST (50 mM Tris; 150 mM NaCl; 0.05% Tween 20)). Primary antibodies were incubated at 4 °C overnight followed by incubation with infrared secondary conjugated antibodies for 1 h at RT. Infrared secondary antibodies were visualised using the the ODYSSEY® Sa Infrared Imaging System (Licor Biosciences, Nebraska, USA).

### 2.9 Structural analysis

Multiple alignment of EPAC1 and EPAC2 sequences (UniProt) and structural analyses were carried out using Jalview [21], Muscle alignment server [22] and PyMOL [23]. Homology models of EPAC1 and EPAC2B were made using Modeller V9.14 [24]. EPAC1 and EPAC2B sequences (UniProt accession numbers, 095398 and A2ASW8, respectively) were aligned with and modelled on EPAC2A (closed conformation, structure 2BYV, [25]) and models with lowest DOPE scores chosen.

## 3 Results

### 3.1 EPAC1 is activated at the nuclei of HEK293T cells following cAMP stimulation

**Fig. 1:**
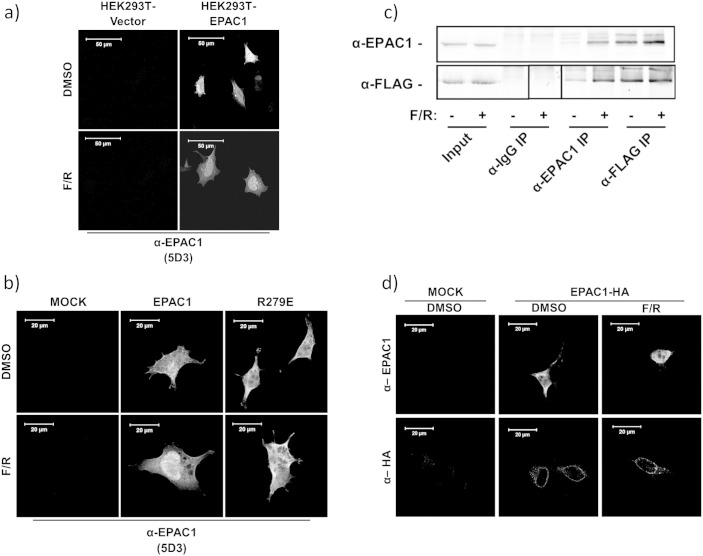
Active EPAC1 is localised to the nucleus in HEK293T cells. a)HEK293T stably expressing either empty vector or EPAC1-FLAG construct was treated with or without a combination of forskolin and rolipram (F/R, 10 μM, 60′). Cells were fixed for immunofluorescence using anti-EPAC1 (5D3) antibodies.b)HEK293T cells were transiently transfected with either wild type EPAC1 or EPAC1-R279E constructs and then incubated with F/R (10 μM, 60′), followed by immuno-detection with anti-EPAC1 (5D3).c)Immunoprecipitation of EPAC1 from stably transfected HEK293T cells. Cell lysates (input) were immunoprecipitated with anti-IgG (mouse), anti-EPAC1 (5D3) or anti-FLAG antibodies (square indicates lane moved for ease of presentation).d)HEK293T cells were transiently transfected with a EPAC1-HA construct. Cells were then treated with F/R (10 μM, 60′), fixed and probed using anti-EPAC1 (5D3) or anti-HA antibodies as indicated.

The subcellular distribution of Flag-tagged EPAC1 was determined in transfected HEK293T cells by immunofluorescent staining with the anti-EPAC1 (5D3) antibody from New England Biolabs. Anti-EPAC1 (5D3) has been reported to preferentially interact with EPAC1 in the active, cAMP-bound state as its epitope lies within the cyclic nucleotide binding domain (CNBD) of EPAC1 [26]. We found that anti-EPAC1 (5D3) was able to detect EPAC1 protein in cells expressing EPAC1, but not in the control cell transfected with vector alone. However, elevation of intracellular cAMP, induced by treatment of cells with a combination of the adenylate cyclase activator, forskolin, and the type 4 PDE inhibitor, rolipram, (F/R) led to increased EPAC1 immunofluorescence within the nuclei of transfected cells (Fig. la). In order to assess the importance of cAMP binding and activation on the nuclear staining of EPAC1, cells were transiently transfected with a mutant form of EPAC1 (EPAC1-R279E), which is deficient in cAMP binding (Fig. 1b). Consequently, EPAC1-R279E immunostaining was unaffected by elevations in intracellular cAMP, indicating that the anti-EPAC1 (5D3) antibody specifically recognises active, cAMP-bound EPAC1 in the nuclei of HEK293T cells.

To confirm that the anti-EPAC1 (5D3) specifically recognises the active form of EPAC1, immunoprecipitation (IP) experiments were carried out on cells transfected with FLAG-tagged EPAC1 (EPAC1-FLAG) using the anti-EPAC1 (5D3) antibody (Fig. 1c). Results demonstrated that more EPAC1-FLAG protein was precipitated using the anti-EPAC1 (5D3) antibody, following F/R stimulation, whereas anti-FLAG IP of EPAC1 was unaffected by elevations in intracellular cAMP (Fig. 1c). This confirms that the anti-EPAC1 (5D3) antibody specifically recognises the active form of EPAC1 in the nuclei of F/R-stimulated HEK293T cells.

To assess whether EPAC1 translocates to the nucleus following cAMP stimulation, we next compared the subcellular distribution of HA-tagged EPAC1 (EPAC1-HA) in transfected HEK293T cells, using anti-EPAC1 (5D3) and anti-HA antibodies (Fig. 1d). Immuno-staining of transfected cells with anti-HA antibodies revealed a distinct perinuclear distribution of EPAC1 (Fig. 1d), which is in agreement with published results [17]. Furthermore, F/R stimulation did not alter the distribution of EPAC1 distribution when probed with an anti-HA antibody, indicating that nuclear EPAC1 protein levels remain constant following stimulation with cAMP. Together these results demonstrate that EPAC1 is pre-localised to the nucleus in HEK293T cells, where it becomes activated following elevations of intracellular cAMP.

### 3.2 EPAC1 and EPAC2A are localised to distinct subcellular compartments

**Fig. 2:**
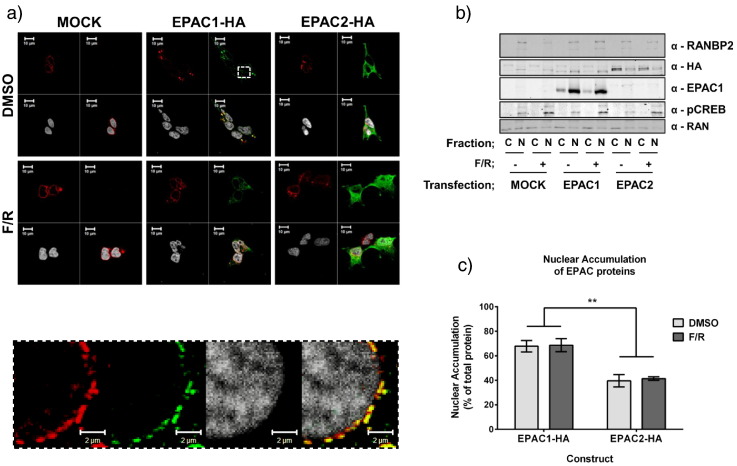
EPAC isoforms display differential targeting in HEK293T cells. a)The subcellular distribution of EPAC1-HA and EPAC2A-HA in HEK293T by immunostaining with anti-HA antibodies. EPAC-HA proteins are stained green, the nuclear pore protein, RanBP2, is stained red and the Reddot nuclear stain is white. Merged images are also shown (EPAC1/RanBP2 colocalisation—yellow). Inset indicates the strong colocalisation of EPAC1 with RanBP2 at the nuclear membrane.b)HEK293 cells were stimulated in the presence or absence of F/R (10 μM, 1 h) and then fractionated into nuclear and cytoplasmic fractions, which were immunoblotted with the indicated antibodies.c)Accumulation of transfected EPAC proteins in the nuclear fraction was assessed by densitometric analysis (n-3, ± s.e.m.). **P < 0.01 (two way ANOVA).

EPAC1 and EPAC2A have been reported to occupy distinct subcellular compartments, whereby EPAC1 displays a mainly perinuclear distribution during interphase [8,16–19] and EPAC2A is largely cytoplasmic [15,27]. We therefore compared the subcellular distribution of EPAC isoforms in HEK293T cells by transfecting cells with EPAC1-HA or EPAC2A-HA constructs and observing their subcellular distribution using immunofluorescent confocal microscopy (Fig. 2a). In agreement with previous studies, EPAC1 was observed to accumulate at the nuclear membrane, whereas EPAC2A was evenly distributed throughout the cell [17,27]. Furthermore, co-staining of EPAC1 with the nuclear pore protein RanBP2 revealed a strong level of co-localisation between EPAC1 and the nuclear pore complex (Fig. 2a, inset). This co-localisation was not observed in EPAC2A transfected cells.

In order to confirm the nuclear distribution of EPAC1, transfected cells were fractionated into nuclear and cytoplasmic components. The fidelity of nuclear preparations was confirmed by immunoblotting for the nuclear pore protein, RanBP2, which is present in nuclear, but not cytosolic fractions (Fig. 2b). Immunoblotting with anti-Ran GTPase demonstrated equal protein loading of nuclear and cytosolic fractions (Fig. 2b). Antibodies towards active, phosphorylated form of the PKA substrate, CREB (Ser 133), were used to demonstrate that F/R treatment promoted and increase in intracellular cAMP. Detection of EPAC1 and EPAC2A within each fraction confirmed the protein distributions revealed by immunofluorescent techniques (Fig. 2b). Specifically, EPAC1 was enriched with the nuclear pore protein RanBP2 within nuclear fractions, whereas EPAC2A was enriched in the cytoplasmic fraction (Fig. 2b). Densitrometric analysis of immunoblots revealed that 68% (± 8%) of total cellular EPAC1 was found within the nuclear fraction compared to only 40% (± 8%) of EPAC2A (Fig. 2c). Similar to the distribution of each isoform observed by microscopic analysis, F/R stimulation had no effect on the distribution of either isoform. It is interesting to note that the distribution of both EPAC1 and EPAC2A was not affected by F/R stimulation (Fig. 2b), despite earlier reports of EPAC1 [17] and EPAC2A [27] translocations in response to cAMP.

### 3.3 The catalytic domain of EPAC1 is required for nuclear localisation

**Fig. 3:**
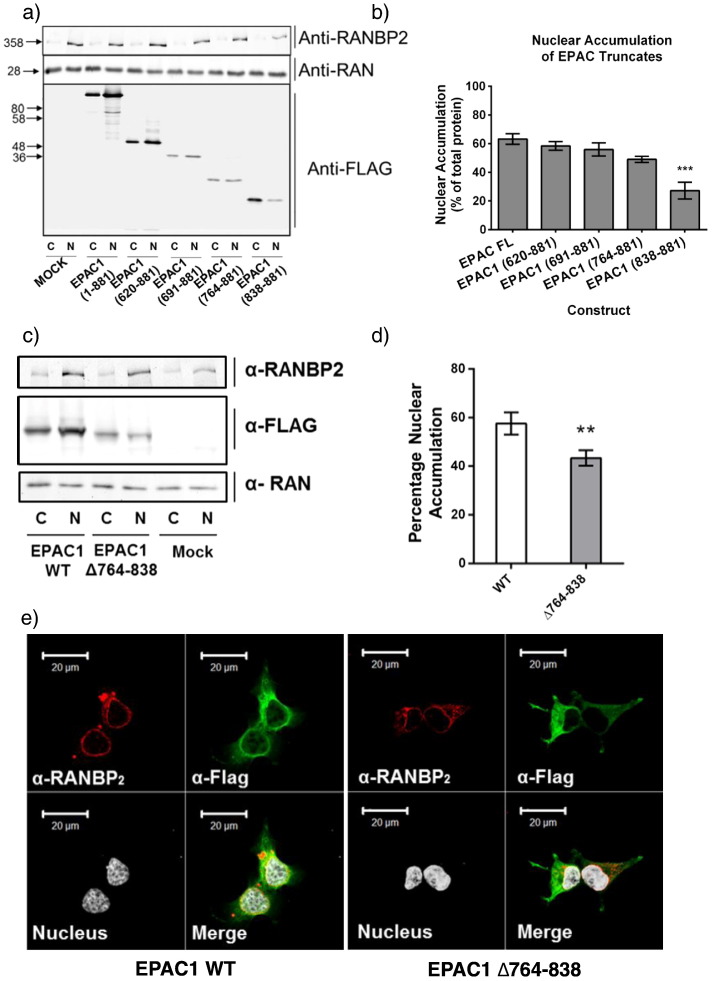
Identification of a nuclear pore targeting signal (NPLS) in EPAC1. a)Fractionation and western blotting of cytoplasmic and nuclear extracts from HEK293T cells transiently transfected with truncated EPAC1 mutants. EPAC FL—full length EPAC1, 620–881 is the full CDC25-HD and 691–881, 764–881 and 838–881 represent further truncations through the CDC25-HD.b)Quantification of the relative distribution of transfected EPAC1 mutants in nuclear fractions (n = 3, ± s.e.m.). ***P < 0.001 (ANOVA).c)Western blot analysis of cytoplasmic and nuclear fractions of HEK293T cells transfected with full length EPAC1 (WT) and mutant EPAC1 lacking amino acids 764–838 (Δ764–838).d)Band intensity was calculated and the percentage of each mutant within the nuclear fraction is shown (n-3, ± s.e.m.) **p < 0.01 (ANOVA).e)HEK293T cells were transfected with EPAC1-WT or EPAC1 Δ764–838 and then immunostained with an anti-FLAG antibody (green) to detect transfected EPAC1. The nuclear membrane was labelled with the anti-RanBP2 antibody (red) and the nucleus highlighted using Reddot nuclear stain (white). Superimposed images reveal co-localisation (merge).

Nuclear accumulation of EPAC1 [10] and interaction with RanBP2 [17] both rely on the catalytic CDC25 Homology Domain (CDC25-HD) of EPAC1, however the precise region within this domain has not been determined. We therefore examined the nuclear localisation of a range of truncated EPAC1 mutants in transfected HEK293T cells (Fig. 3). As previously reported [10,17] deletion of the N-terminal regulatory domain of EPAC1 to the CDC25HD (EPAC1 620–881) had no effect on nuclear accumulation, indicating that the N-terminus of EPAC1 is dispensable for nuclear targeting (Fig. 3a). In order to ascertain the region required for nuclear targeting, the CDC25-HD was further truncated by 70 amino acid increments yielding EPAC1 691–881, 764–881 and 838–881. Both EPAC1 691–881 and EPAC1 764–881 displayed a similar distribution to wild type EPAC1 within the nuclear fraction (Fig. 3a). However, in agreement with our previous observations in COS1 cells [10], EPAC1 838–881 was observed to significantly accumulate within the cytoplasmic fraction, suggesting that nuclear localisation may require amino acids 764–838 (Fig. 3a and b). In order to confirm the importance of these residues in nuclear targeting, amino acids 764–838 were deleted from full length EPAC1. Fractionation of cell extracts from EPAC1 WT and EPAC1 Δ764–838-transfected cells revealed significantly higher levels of cytosolic EPAC1 Δ764–838 compared to wild type protein (Fig. 3c and d), suggesting that these residues are involved in the nuclear localisation of EPAC1. Moreover, immunofluorescent staining demonstrated that EPAC1 Δ764–838 failed to accumulate at the perinuclear domain and adopted a cytosolic distribution in contrast to full length protein (Fig. 3e). Cell fractionation and immunofluorescent detection together implicate amino acids 764–838 as being vital for determining the subcellular distribution of EPAC1 and therefore represents a novel nuclear pore localisation signal (NPLS) in EPAC1.

### 3.4 EPAC1 and EPAC2 share a conserved nuclear pore targeting signal

**Fig. 4:**
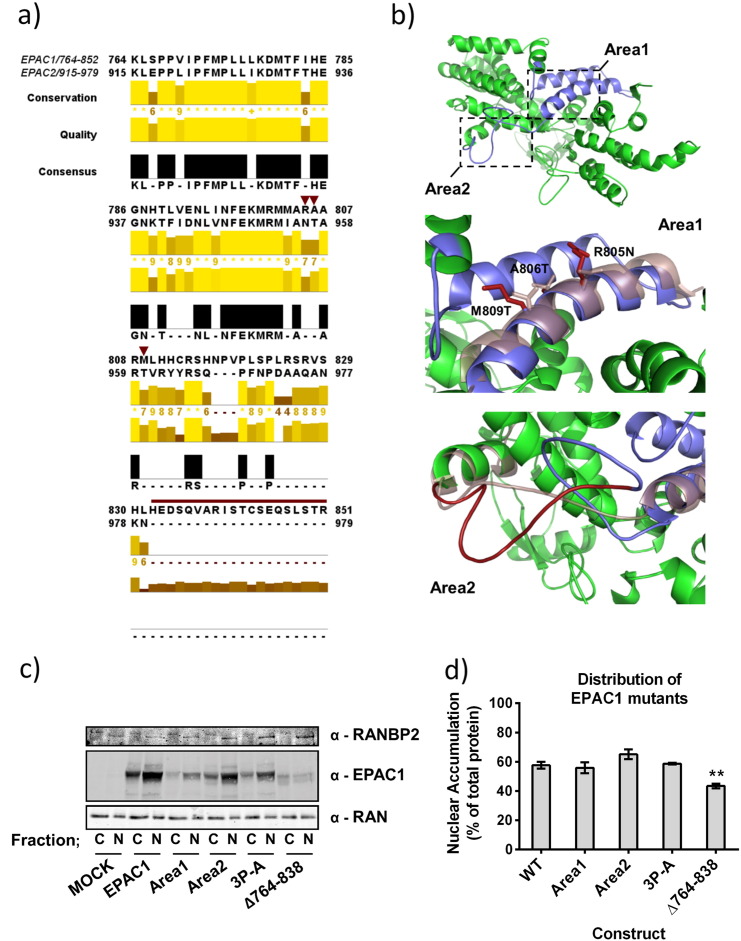
Mutation of non-conserved regions within the EPAC1 NPLS has no effect on EPAC1 distribution. a)Sequence alignment of EPAC1 (a.a. 764–851) and EPAC2 (a.a. 915–979) indicating residues 764–838 (NPLS) and a.a. 839–851 which are absent in EPAC2, but present in EPAC1. This region is highly conserved with the exception of two regions also predicted to be surface exposed when mapped onto a homology model of EPAC1. The positions of Area1 point mutations (R805N, A806T and M809T) are indicated (red arrowheads) and residues deleted by Area2 mutagenesis (a.a. 832–851) are highlighted (red line).b)Homology modelling of EPAC1 based on EPAC2 crystal structure (2byv) [25] shows the CDC25-HD (green) and NPLS (Blue). Insets identify the potential interaction sites, Area1 and Area2, with specific residues of EPAC1 shown (red). The effects of EPAC1 mutagenesis on the corresponding EPAC2A sequences are shown by superimposing the structure of EPAC2A (purple, transparent).c)HEK293T cells were transfected with full length EPAC1 or NPLS mutants designed to disrupt nuclear accumulation. Fractionation and immuno-detection of proteins revealed the accumulation of each mutant in the cytoplasmic or nuclear fractions.d)Band intensity was calculated from western blots and the amount of nuclear EPAC1 was calculated as a percentage of total protein observed (n-3, ± s.e.m.). **p < 0.01 (ANOVA).

Control over nuclear targeting of EPAC1 has been linked to a direct interaction between the EPAC1 CDC25-HD and RanBP2 [17]. Our work identifies a region within the CDC25-HD that is involved in nuclear targeting (Fig. 3). Since EPAC1 is targeted to the nucleus by this NPLS, whereas EPAC2A is not (Fig. 3), regions of low sequence similarity within the NPLS may reveal structural differences that underlie the differential targeting of EPAC1 and EPAC2A. We therefore aligned the primary sequence of EPAC1 764–851 with the corresponding region of EPAC2A (amino acids 915–979). The CDC25-HD and the NPLS are intimately involved in GEF activity and interactions with Rap GTPase and as such EPAC1 and EPAC2A share a considerable sequence similarity within this region (Fig. 4a). However, two regions of low sequence similarity, dubbed Area1 and Area2, were identified that may underlie the differences in localisation observed between EPAC1 and EPAC2A (Fig. 4a). In addition to low sequence similarity, both sites were determined to be available for protein–protein interaction by their exposure to solvent (based on EPAC1 homology model, Fig. 4b). Area1 (composing residues R805, A806 and M809, EPAC1 nomenclature) is found within a groove between two conserved alpha helices and may form a suitable site for protein interaction (Fig. 4b). Area2 is a region of 20 amino acids which forms an extended loop that is absent in EPAC2A and sufficiently long to afford EPAC1 a unique protein–protein interaction motif (Fig. 4b). As a result of their low sequence similarity and availability for protein–protein interaction, Area1 and Area2 represent good candidate regions involved in EPAC1 nuclear targeting.

In order to test the importance of Area1 and Area2, their amino acid sequences were converted by mutagenesis to the corresponding region of EPAC2A, i.e. Area 1 (R805N, A806T, and M809T) and Area 2 (Δ832–851). As EPAC2A displays a mainly cytoplasmic distribution, mimicking the sequence of EPAC2A within these regions may disrupt nuclear localisation of EPAC1 while maintaining catalytic activity. In addition, mutagenesis of a rigid loop between Area1 and Area2 was designed to disrupt both the secondary structure and protein–protein interactions within this region of EPAC1. Accordingly, EPAC1 P819A, P821A, P824A (3P-A) releases the rigidity imparted by each proline residue which may define the structure of the region between Area1 and Area2. Fractionation of transfected HEK293T cells revealed that while Δ764–838 was enriched within the cytoplasmic fraction, Area1, Area2 and 3P-A mutagenesis did not have any significant effect on the subcellular distribution of EPAC1 (Fig. 4c and d). These data suggest that the difference in localisation observed between EPAC1 and EPAC2A is not due to interactions involving Area1 or Area2 of the EPAC1 NPLS.

### 3.5 CNBD1 of EPAC2 disrupts nuclear localisation

**Fig. 5:**
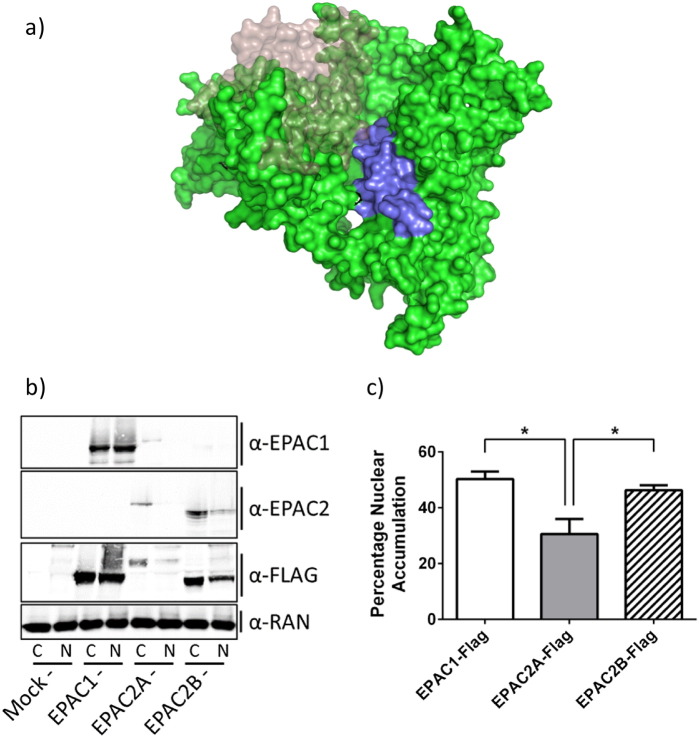
EPAC2B is more enriched in nuclear fractions than EPAC2A. a)Homology model of EPAC2B (based on sequence alignment of EPAC2B with EPAC2A, crystal structure 2BYV) is shown (green). The additional N-terminal CNBD1 of EPAC2A is superimposed (transparent) to highlight its proximity to the NPLS (purple).b)HEK293T cells were transfected with FLAG-tagged EPAC1, EPAC2A or EPAC2B. Fractionation and immunoblotting revealed the distribution of each protein within the cytosolic and nuclear fractions.c)Quantitative densitometry and calculation of nuclear EPAC were carried out for each transfected protein (n-3, ± s.e.m.). *p < 0.05 (ANOVA).

The NPLS of EPAC1 appears to be important for nuclear targeting of EPAC1. However this region is strongly conserved in EPAC2A, which has reduced nuclear targeting. This observation suggests that structural differences between EPAC1 and EPAC2A may underlie the differences in the localisations observed. One major difference between the two isoforms is the presence of a second, N-terminal CNBD (CNBD1) in EPAC2A. Furthermore, CNBD1 has previously been implicated in the subcellular targeting of EPAC2A [15]. Interestingly, a third, tissue specific isoform of EPAC has been recently identified in the adrenal glands [15]. In this case, differential splicing of the EPAC2A gene results in the loss of the additional N-terminal CNBD1, yielding a truncated form, EPAC2B [15]. Interestingly, comparison of an EPAC2B homology model and the crystal structure of EPAC2A (2BYV [25]) reveals that CNBD1 lies in close proximity to the N-terminal section of the NPLS in EPAC2A (Fig. 5a). It is therefore possible that steric interference from CNBD1 blocks access of potential nuclear localisation partners, such as RanBP2, to the NPLS of EPAC2A, but not EPAC1 or EPAC2B. In order to assess the importance of the CNBD1 of EPAC2A in nuclear localisation, EPAC1, EPAC2A and EPAC2B were transfected into HEK293T and their sub-cellular distributions assessed by nuclear fractionation (Fig. 5b). Results demonstrated that while EPAC2B was observed to accumulate within the nuclear fraction, the full length EPAC2A construct was largely cytosolic. Interestingly, EPAC2B was observed to be distributed between the nuclear and cytoplasmic fractions (approximately 50:50), similar to EPAC1 (Fig. 5b, c). These data support the idea that CNBD1 is an important factor in determining the subcellular distribution of EPAC2A.

## 4 Discussion

Various reports have demonstrated localisation of EPAC1 and EPAC2A to distinct subcellular locales [15,17,19,27]. Indeed, we have confirmed that EPAC1 is tethered to the nucleus in transfected HEK293T cells, whereas EPAC2A is largely cytosolic (Fig. 1). We identified a nuclear pore localisation signal (NPLS; amino acids 764–838) within the catalytic domain of EPAC1 that appears to be responsible for nuclear tethering. We propose here that the CNBD1 of EPAC2A may block access to the corresponding NPLS of EPAC2A, reducing its ability to interact with nuclear binding sites. Indeed, a naturally occurring EPAC2 isoform lacking CNBD1, EPAC2B, exhibits significant enrichment within the nuclear fraction, similar to EPAC1. Our structural models suggest that the reason for this is that the NPLSs of EPAC1 and EPAC2B are more exposed for protein–protein interactions with nuclear pore complex (NPC) proteins.

In this regard, it has previously been shown that RanBP2 interacts directly with the CDC25-HD of EPAC1 to mediate its localisation to the nuclear envelope [17]. We have, however, been unable to demonstrate this interaction in our cell system (results not shown), which suggests that interactions with other protein constituents of the NPC may facilitate EPAC1 recruitment in HEK293T cells. In support of this idea is our observation that EPAC1 becomes activated following elevations in intracellular cAMP in HEK293T cells (Fig. 1), whereas previous work has shown that interactions with RanBP2 inhibit EPAC1 activity at the nuclear pore. This suggests that the interactions responsible for NPC recruitment in HEK293T cells do not suppress EPAC1 activity and may therefore exert novel regulatory control over the actions of nuclear EPAC1. Nuclear tethering of EPAC1 may therefore determine its ability to be activated by cAMP in a cell type-specific manner, dependent on whether or not EPAC1 has access to interaction with RanBP2 or other binding proteins that do not inhibit activity. This also suggests that in the future, chemical modulators could be devised that specifically disrupt nuclear targeting, and hence the activity of EPAC1 and EPAC2B, without affecting the activity of EPAC2A. This type of isoform-specific regulation might be important due the differential roles each isoform are observed to play *in vivo*. For example, EPAC1 is the principal isoform in vascular endothelial cells, where it mediates anti-inflammatory signalling [28], whereas EPAC2A is involved in incretin-stimulated insulin secretion in pancreatic β-cells [29–31]. Therefore it may be possible to design drugs to regulate specific EPAC actions in one tissue while sparing another, thereby reducing the potential risk of side effects.

## References

[1] Stangherlin A., Zaccolo M. (2012). Heart Circ. Physiol..

[2] Buxton I.L., Brunton L.L. (1983). J. Biol. Chem..

[3] Scott J.D., Dessauer C.W., Tasken K. (2013). Annu. Rev. Pharmacol. Toxicol..

[4] Taylor S.S., Zhang P., Steichen J.M., Keshwani M.M., Kornev A.P. (2013). Biochim. Biophys. Acta.

[5] Taylor S.S., Kim C., Vigil D., Haste N.M., Yang J., Wu J., Anand G.S. (2005). Biochim. Biophys. Acta.

[6] de Rooij J., Zwartkruis F.J., Verheijen M.H., Cool R.H., Nijman S.M., Wittinghofer A., Bos J.L. (1998). Nature.

[7] Kawasaki H., Springett G.M., Mochizuki N., Toki S., Nakaya M., Matsuda M., Housman D.E., Graybiel A.M. (1998). Science (New York, N.Y.).

[8] Qiao J., Mei F.C., Popov V.L., Vergara L.A., Cheng X. (2002). J. Biol. Chem..

[9] Mei F.C., Cheng X. (2005). Mol. Biosyst..

[10] Borland G., Gupta M., Magiera M.M., Rundell C.J., Fuld S., Yarwood S.J. (2006). Mol. Pharmacol..

[11] Yarwood S.J. (2005). Biochem. Soc. Trans..

[12] Gupta M., Yarwood S.J. (2005). J. Biol. Chem..

[13] Magiera M.M., Gupta M., Rundell C.J., Satish N., Ernens I., Yarwood S.J. (2004). Biochem. J..

[14] Sehrawat S., Cullere X., Patel S., Italiano J., Mayadas T.N. (2008). Mol. Biol. Cell.

[15] Niimura M., Miki T., Shibasaki T., Fujimoto W., Iwanaga T., Seino S. (2009). J. Cell. Physiol..

[16] Mei F.C., Qiao J., Tsygankova O.M., Meinkoth J.L., Quilliam L.A., Cheng X. (2002). J. Biol. Chem..

[17] Gloerich M., Vliem M.J., Prummel E., Meijer L.A., Rensen M.G., Rehmann H., Bos J.L. (2011). J. Cell Biol..

[18] Liu C., Takahashi M., Li Y., Dillon T.J., Kaech S., Stork P.J. (2010). Mol. Cell Biol..

[19] Wang Z., Dillon T.J., Pokala V., Mishra S., Labudda K., Hunter B., Stork P.J. (2006). Mol. Cell. Biol..

[20] Ross S.H., Post A., Raaijmakers J.H., Verlaan I., Gloerich M., Bos J.L. (2011). J. Cell Sci..

[21] Waterhouse A.M., Procter J.B., Martin D.M., Clamp M., Barton G.J. (2009). Bioinformatics.

[22] Edgar R.C. (2004). Nucleic Acids Res..

[23] DeLano W.L. (2002). PyMOL molecular graphics system. http://www.pymol.org.

[24] Sali A., Blundell T.L. (1993). J. Mol. Biol..

[25] Rehmann H., Das J., Knipscheer P., Wittinghofer A., Bos J.L. (2006). Nature.

[26] Zhao J. (2006). Biologie Proefschriften.

[27] Li Y., Asuri S., Rebhun J.F., Castro A.F., Paranavitana N.C., Quilliam L.A. (2006). J. Biol. Chem..

[28] Parnell E., Smith B.O., Palmer T.M., Terrin A., Zaccolo M., Yarwood S.J. (2012). Br. J. Pharmacol..

[29] Dzhura I., Chepurny O.G., Leech C.A., Roe M.W., Dzhura E., Xu X., Lu Y., Schwede F., Genieser H.G., Smrcka A.V., Holz G.G. (2011). Islets.

[30] Kelley G.G., Chepurny O.G., Schwede F., Genieser H.G., Leech C.A., Roe M.W., Li X., Dzhura I., Dzhura E., Afshari P., Holz G.G. (2009). Islets.

[31] Shibasaki T., Takahashi H., Miki T., Sunaga Y., Matsumura K., Yamanaka M., Zhang C., Tamamoto A., Satoh T., Miyazaki J., Seino S. (2007). Proc. Natl. Acad. Sci. U. S. A..

